# lncRNA TUSC7 regulates oxidative stress level by targeting miR-23b in colorectal cancer and thus inhibits cell proliferation, migration and invasion

**DOI:** 10.18632/aging.205270

**Published:** 2023-12-05

**Authors:** Haopeng Ye, Weidan Ren, Guiwei Liu, Qingjin Guo

**Affiliations:** 1 Departments of Colorectal and Anal Surgery, Cang Zhou Central Hospital, Cang Zhou 061000, Hebei, China

**Keywords:** colorectal cancer, lncRNA TUSC7, miR-23b, oxidative stress, cell proliferation

## Abstract

Objective: Currently, multiple studies have shown that long non-coding ribonucleic acid TUSC7 exerts an anti-tumor effect in a variety of cancers. However, the function and underlying regulatory mechanism of lncRNA TUSC7 in CRC remain unclear.

Methods: The relative fluorescence intensity of MMP9 in the cancer and in peritoneal tissues was measured by immunofluorescence. Peritoneal macrophages in BALB/c mice were sorted out using flow cytometry. The abdominal circumference of mice was measured. Moreover, the correlation between TUSC7 and miR-23b was detected by diluciferase experiment and the expressions of TUSC7 and miR-23b were analyzed using real-time fluorescence quantitative PCR. Last, the effect of TUSC7 on peritoneal macrophages was detected.

Results: The relative fluorescence intensity of MMP9 in cancer was significantly stronger than that it in the surrounding tissues. Measurements of abdominal circumference in mice showed that TUSC7 inhibited the metastasis of CRC. The results of dual luciferase assay and RT-qPCR experiment showed that TUSC7 could target and inhibit miR-23b. The expressions of P22, P47, gp91, p-STAT6, p-STAT3, IL-4 and IL-10 were remarkably increased in TUSC7 OE group compared with those in NC group, while the expressions of p-SHP2, MMP2 and MMP9 were evidently reduced in contrast with those in NC group. The viability, proliferation, migration and invasion of CRC cells could be inhibited by TUSC7 OE, which was reversed by TUSC7 KD.

Conclusion: LncRNA TUSC7 can regulate the oxidative stress level and promote the M2 polarization of macrophages through targeting miR-23b of peritoneal macrophage in CRC, thus inhibiting cell proliferation, migration and invasion.

## INTRODUCTION

Colorectal cancer (CRC) is the third most common malignancy and the second leading cause of cancer-related death worldwide. When CRC, particularly advanced CRC, is diagnosed, patients usually experience symptoms such as anemia [1, 2]. According to relevant statistics, the incidence of anemia in patients with gastrointestinal cancer is 51.13% in China, more than 90% of whom receive no corrective treatment for anemia before malignancy is diagnosed. Numerous studies have confirmed the association between anemia and poor prognosis in CRC patients [3, 4].

Long non-coding ribonucleic acids (lncRNAs) are a class of endogenous RNAs without protein-coding potential. The research of LncRNAs is the focus of molecular biology, and the field has advanced rapidly. LncRNAs can regulate their downstream genes and participate in almost all cell biological phenotypes, such as cell cycle, apoptosis, autophagy and drug resistance. Studies have indicated that lncRNAs are expressed aberrantly in almost all human malignancies, and they are involved in tumorigenesis as oncogenes or tumor suppressor genes [5–8]. The antisense RNA tumor suppressor candidate 7 (TUSC7), which consists of four exons and is located at chromosome 3q13.3, is classified as a long non-coding RNA (lncRNAs) because it has no potential to encode proteins [9, 10]. Recently, it has been found that TUSC7 is down-regulated and serves as a potential tumor suppressor gene in several malignancies, including CRC, non-small cell lung cancer, hepatocellular carcinoma and gastric cancer. However, the mechanism of TUSC7 in CRC is poorly understood [11, 12].

Studies have shown that micro RNA (miR)-23b can suppress oxidative stress in tumors, whose downstream target gene is phosphatase and tensin homolog deleted on chromosome ten (PTEN), and exosomal miR-23b also exerts its antioxidant effect by regulating the PTEN/Nrf2 pathway [13, 14]. In addition, miR-23b reduces the binding of PTEN to the NOD-like receptor family pyrin domain containing 3 (NLRP3) inflammasome, thereby reducing the level of NLRP3-dependent pyroptosis. When TUSC7 binds to miR-23b like a sponge, miR-23b is rendered inactive, which indirectly regulates oxidative stress levels in tumors [15–18].

In this study, the expression levels of TUSC7 and miR-23b were analyzed to investigate their regulatory effects and molecular mechanisms on related proteins in CRC. The results manifested that TUSC7 could promote oxidative stress by inhibiting miR-23b, thereby contributing to M2 polarization of macrophages through the SH2 domain-containing tyrosine phosphatase-2 (SHP2)-signal transducer and activator of transcription 3 (STAT3)-STAT6 axis and suppressing proliferation, migration and invasion of CRC cells.

## MATERIALS AND METHODS

### Animal modeling

Male BALB/c mice (purchased from Jackson Laboratory) and colon adenocarcinoma CT26 cell lines (Procell) were used. CT26 cells were routinely cultured with Roswell Park Memorial Institute (RPMI)-1640 medium containing 10% fetal bovine serum (FBS). The cells in the exponential growth phase were collected, digested, and pipetted into single-cell suspension, then centrifuged at 1,000 r/min for 5 min. After the supernatant was discarded, the cell concentration was adjusted to 1 × 10^6^ cells/mL using an appropriate amount of serum-free RPMI-1640 medium. Finally, 0.2 mL of CT26 cell suspension (1 × 10^6^/mL) was slowly injected into the right of midline of the lower abdomen of BALB/c mice. The abdominal circumference of mice was then observed and measured.

### Flow cytometry

Tissues aseptically harvested were washed with D-phosphate buffered saline (PBS) to remove blood stains, and cut into 1 × 3 mm blocks with sterile scissors. The tissue blocks were stirred in a solution containing 0.2% collagenase type IV (Sigma_Aldrich, USA) and 0.25% trypsin (Thermo Fisher, USA) for 5 min at 37°C [19]. After termination of digestion, the supernatant was harvested, filtered through a 200-micron mesh, resuscitated with staining buffer (1 × PBS containing 1% BSA), and stored at 4°C. Cell staining and flow sorting were then conducted. After the cell density was adjusted to 1 × 10^6^/100 μL, the cells were stained with CD68 Monoclonal Antibody (Ki-M7), FITC, Goat anti-Mouse IgG1 Cross-Adsorbed Secondary Antibody and Alexa Fluor™ 594 away from light for 30 min. Then the cells were analyzed and sorted with a BD FACSAria III sorting flow cytometer (BD Biosciences, USA), and the sorted macrophages were used in subsequent assays.

### Immunofluorescence staining

The prepared sample was taken out, cooled to room temperature, and washed three times with PBS for 5 min each time. Then, the sample was soaked in the citric acid buffer, and repaired with low heat in the microwave oven. After cooling, the sample was washed three times with PBS for 5 min each time and blocked for 20 min. The first antibody was the diluted to the target ratio with antibody diluent at 4°C overnight. After rewarmed for 30 min, the fist antibody was rinsed three times with PBS for 5 min each time. The fluorescent secondary antibody was diluted with antibody diluent, incubated for 2 h at room temperature away from light. Then it was eluted with PBS three times for 5 min each time. Then, the nucleus was stained with anti-fluorescence quenching blocking solution and the tablet was sealed and kept at 4°C away from light. Last, the fluorescent image was captured after the fluorescence microscope observation.

### Cell transfection and culture

The overexpression (OE) vector pE-TUSC7 and blank vector pEGFP-N1 (Genescript, Nanjing, China) were used. To inhibit TUSC7, 4 small interfering RNAs (siRNAs) (TUSC7-siRNA1, TUSC7-siRNA2, TUSC7-siRNA3 and TUSC7-siRNA4; Gene Pharmaceuticals, Shanghai, China) were synthesized and optimized. Upon reaching approximately 60–70% confluency, the cells were transfected with Lipofectamine™ 3000 reagent. Then the cells were divided into negative control (NC) group, TUSC7 OE group and TUSC7 KD group. Mouse macrophages were cultured with Dulbecco’s modified Eagle medium (DMEM) supplemented with 10% FBS, 100 U/mL streptococci, non-essential amino acids (NEAA), and 100 U/mL streptomycin in a 5% CO_2_ humid chamber at 37°C, and then incubated with LPS (1 μg/mL) overnight at 37°C. Finally, the supernatants and cell lysates were collected and stored at −70°C for further analysis.

### Real-time quantitative polymerase chain reaction (RT-qPCR)

To quantify TUSC7 and miR-26b, total RNA was extracted using TRIzol and reversely transcribed into cDNA using the Reverse Transcription Kit (TaKaRa, Bio, USA). Then real-time quantitative PCR was performed using the SYBR-Green PCR Master Mix Kit (TaKaRa Bio, USA). The gene expression level was calculated using the 2^−ΔΔCt^ method. The primer sequences are as follows: TUSC7 (F: 5′-TTTATGCTTGAGCCTTGA-3′; R: 5′-CTTGCCTGAAATACTTGC-3′), miR-26b (F: 5′-GCGGTCATCACATTGCCAG-3′; R: 5′-GCGGTCATCACATTGCCAG-3′), and glyceraldehyde-3-phosphate dehydrogenase (GAPDH) (F: 5′-TGAAGGGTGGAGCCAAAAG-3′; R:5′-AGTCTTCTGGGTGGCAGTGAT-3′).

### Dual luciferase assay

A double luciferase reporter plasmid was constructed using Genescript (Nanjing, China), including pmiR-TUSC7-wt (containing miR-23b target binding sequence in TUSC7) and pmiR-MACC1-mut (containing mutational binding sequence). The dual luciferase assay is performed by co-transfection of pmiR-TUSC7-wt or pmiR-MACC1-mut and agomir-23b or agomir-NC into peritoneal macrophages. Luciferase activity after 48 h of co-transfection of peritoneal macrophages was detected with the Dual Luciferase Reporter Detection System (Promega, USA) in accordance with the manufacturer’s protocol.

### Western blotting

The protein was extracted from cells using lysis buffer (Solarbio, Beijing, China). By 12% sodium dodecyl sulfate-polyacrylamide gel electrophoresis (SDS-PAGE), the protein was transferred onto polyvinylidene fluoride (PVDF) membranes (Millipore, USA), and then the membranes were incubated with primary antibodies against P22, P47, gp91, phosphorylated (p)-SHP2, t-SHP2, p-STAT3, p-STAT6, interleukin-4 (IL-4), IL-10, matrix metalloproteinase-2 (MMP2), MMP9 and GAPDH overnight at 4°C. After washed with TBST, the membranes were incubated with horseradish peroxidase-labeled secondary antibodies at room temperature for 2 h, and detected using an enhanced chemiluminescence detection system (Bio-Rad, USA) according to the manufacturer’s protocol.

### Cell co-culture

Peritoneal macrophages in each group were seeded into cross-well chambers and cultured for 24 h. CT26 cells and CMT93 cells were seeded in culture plates and cultured for 24 h. After incubation overnight, the medium in the chambers and plates was removed and a new culture medium was added to the lower chamber. Then, the Transwell chamber was placed in the culture plate for 48 h, and then new medium was added for detection. The cross-well chamber was removed and the medium was aspirated from the culture plate. Finally, the cells were fixed with 4% paraformaldehyde and washed twice with PBS.

### Transwell assay

The migration and invasion capacity of cells was detected *in vitro* using Transwell chambers with polycarbonate membranes (251.87 mm in diameter, a pore size of 6 μm) (Costar Corning, USA). In the migration assay, the transfected cells were resuspended in 10 μL of serum-free medium at a density of 100 × 5/mL and added to the upper chamber. Next, 12 μL of DMEM supplemented with 10% FBS was added to the lower chamber. After incubation for 20 h, the cells on the upper membrane surface were removed, and the migrating cells were fixed with methanol and stained with 80% Giemsa stain solution. Finally, the stained cells were counted microscopically in five randomly selected fields, and the average was taken. In the invasion assay, the Transwell membranes were applied to 500 μL of Matrigel solution (37 ng/μL, BD Biosciences, USA), and the cells were incubated at 37°C for 4 h. The remaining steps were the same as those in the migration assay.

### Monoclonal formation assay

After 48 h of transfection, the cells were collected, digested with trypsin and counted. They were cultured in an incubator at 37°C until visible cell colonies were formed. Then the medium was discarded, and the cells were washed with PBS 3 times. Afterwards, the cells were fixed with methanol for 15 min, then dried and stained with crystal violet for 30 min. After drying, the cells were scanned and photographed, and the visible colonies were counted.

### Cell counting kit-8 (CCK8) assay

After 48 h of culture, 10 μL of CCK8 reagent was added to each well and incubated for 2 h. Then the optical density (OD) at 450 nm was measured with a microplate reader to determine the cell viability.

### Data analysis

Statistical analysis was performed using SPSS 22.0 software and processed with GraphPad Prism 5.0. Measurement data of normal distribution were expressed by mean ± standard deviation ($\bar{x}\pm \text{s}$). Independent-sample *t*-test and one-way analysis of variance were used for comparison between two groups and among multiple groups, respectively. *P* < 0.05 or *p* < 0.01 was considered statistically significant.

## RESULTS

### The metastasis in cancer is stronger than the tissues around cancer

**Figure 1:**
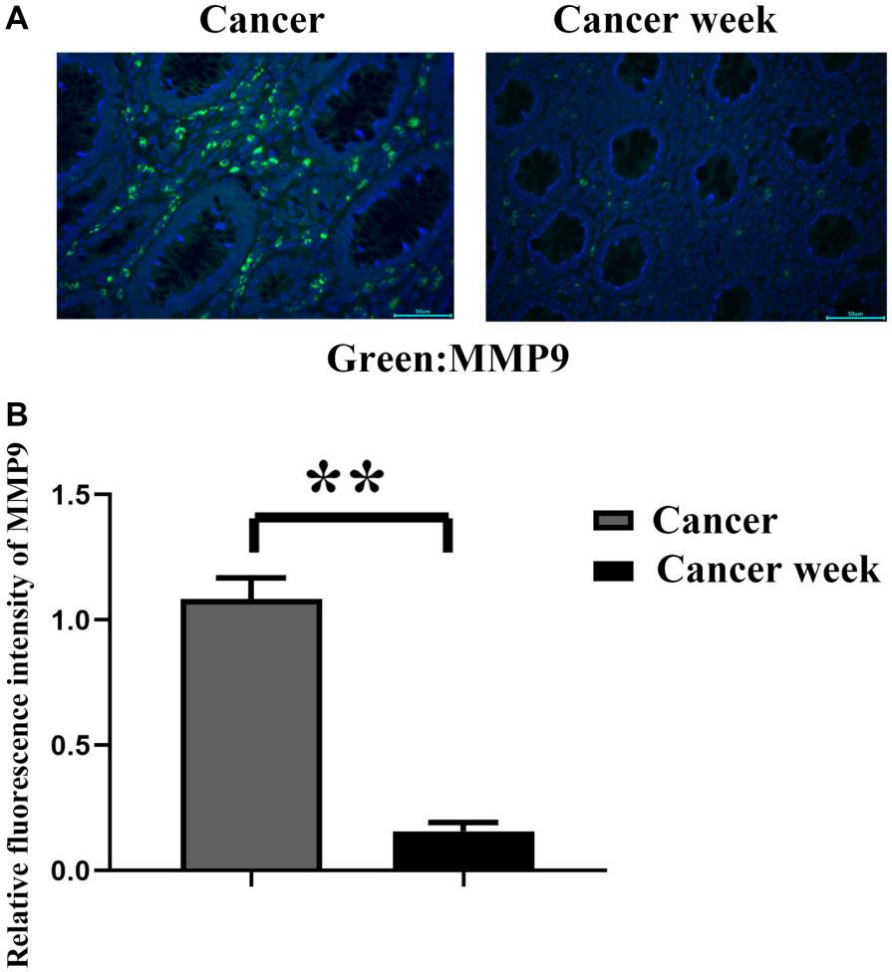
**The relative fluorescence intensity of MMP9 in cancer and in peritoneal tissues was detected by immunofluorescence staining.** (**A**) The graph of immunofluorescence results; (**B**) Statistics for relative fluorescence intensity of MMP9 in cancer and in its peritoneal tissues. ^**^p < 0.01.

By immunofluorescence staining, we found that the relative fluorescence intensity of MMP9 in human cancer was significantly stronger than that in the peritoneal tissues. It indicated that the metastatic ability in cancer was stronger than that in the tissues around cancer. Therefore, the study on the cancer itself is more convincing than that on the cancerous tissues that surround it (Figure 1).

### TUSC7 can inhibit the metastasis of CRC cells to the peritoneum

**Figure 2:**
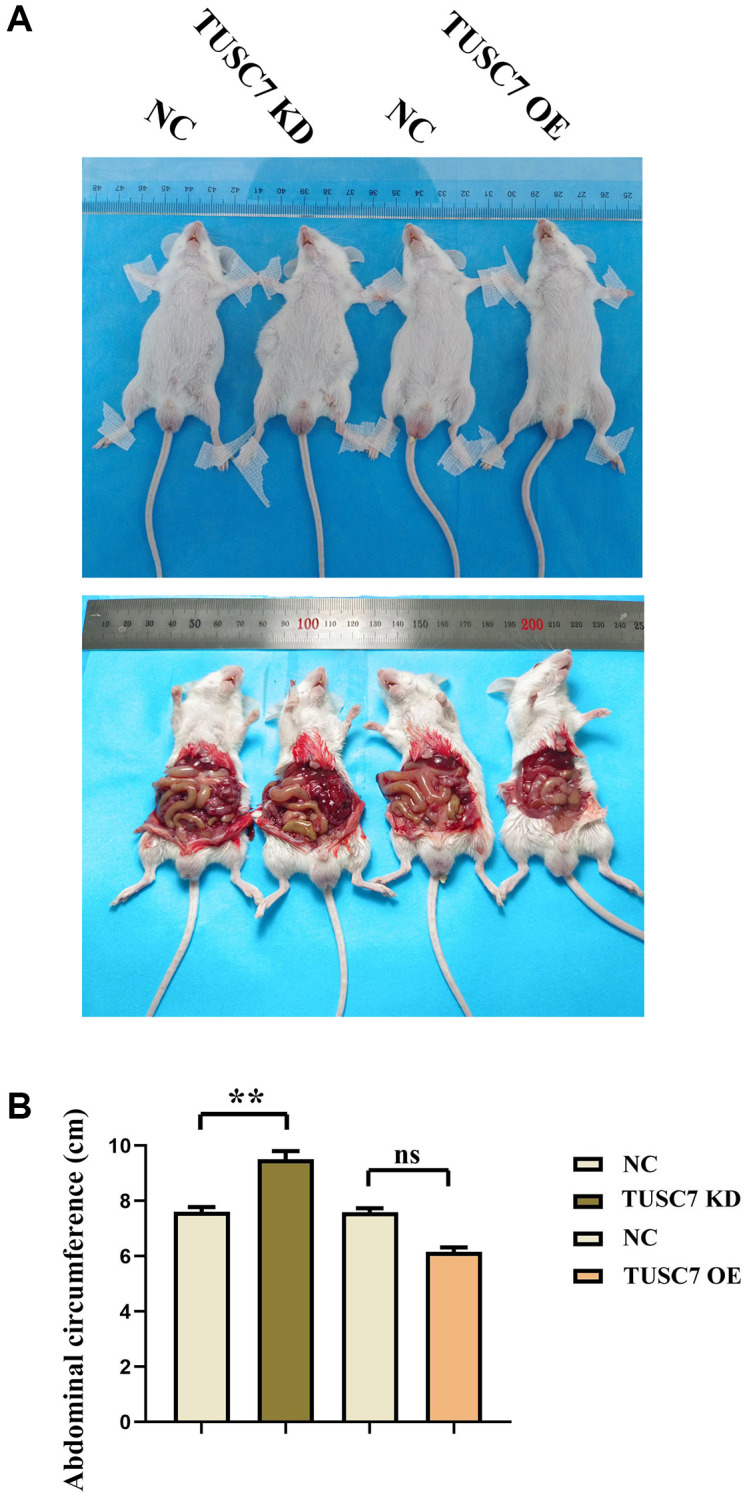
**TUSC7 can inhibit the transfer of CRCs.** (**A**) Mouse CRC peritoneal transfer results plot. (**B**) Statistics of mouse abdominal circumference measurement data. ^**^p < 0.01.

By measuring the abdominal circumference of mice in each group, we found that the abdominal circumference of mice in the TUSC7 KD group increased significantly compared with the NC group. The abdominal circumference of mice in the TUSC7 OE group was significantly reduced. It shows that TUSC7 could restrain the metastasis of CRC (Figure 2).

### TUSC7 can inhibit the expression of miR-23b

**Figure 3:**
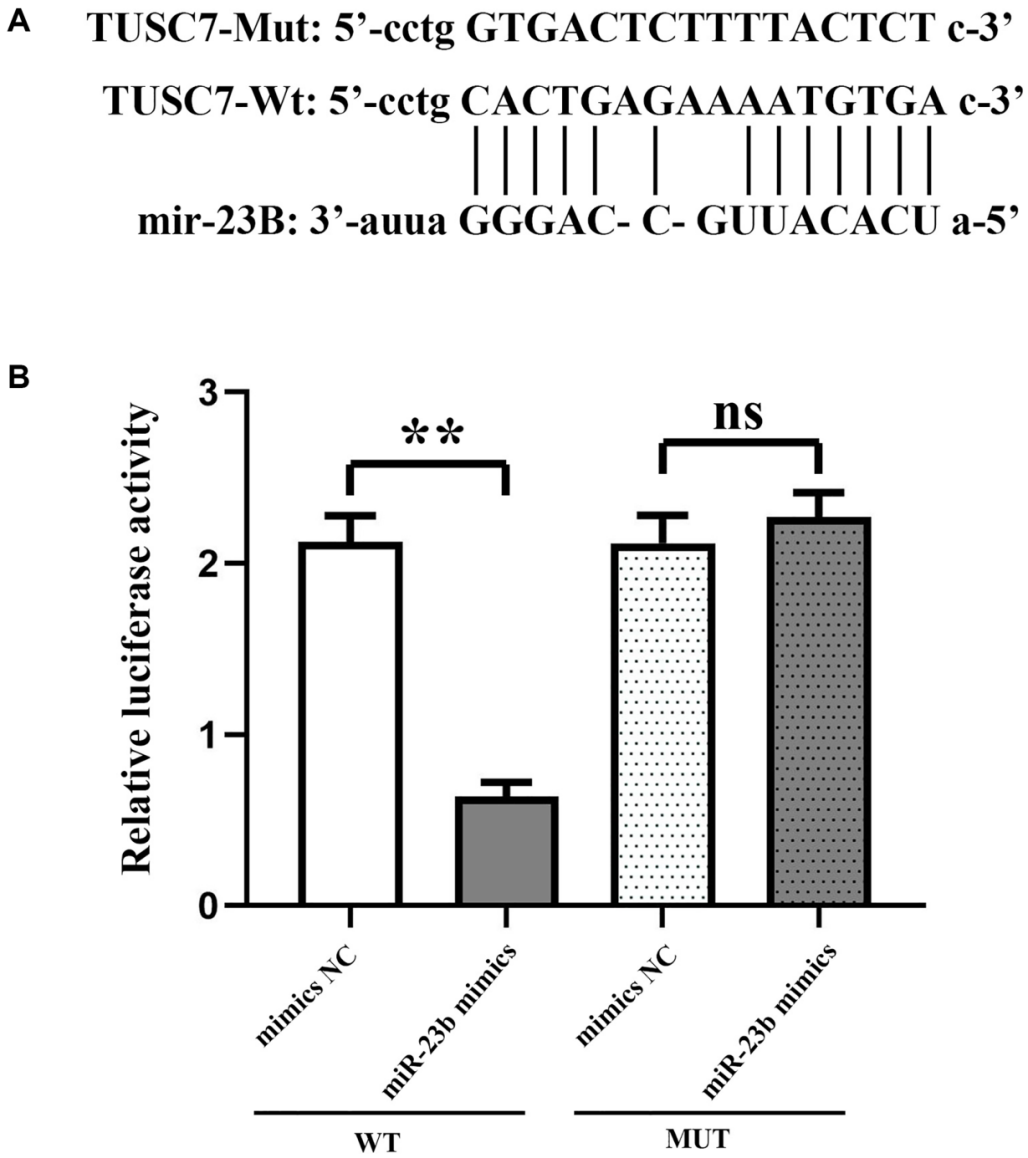
**TUSC7 specifically inhibits miR-23b.** (**A**) Putative binding sites of miR-23b within TUSC7 mRNA, and the sequences of wild-type and mutant-type vectors. (**B**) In peritoneal macrophages co-transfected with wild-type vectors and pagomir-23b, relative luciferase activity was inhibited rather than co-transfected with mutant vectors. Firefly luciferase activity normalized to renilla luciferase. ^**^p < 0.01.

Bioinformatics analysis was used to search for potentially targeted microRNAs for TUSC7, such as Targetcan, Starbase. A miR-23b binding site (1125-1140 bp) was identified in the transcript of TUSC7, the predictor gene for miR-23b. The double luciferase reporter gene showed that by enhancing miR-23b expression, the luciferase activity of wild-type vector-transfected peritoneal macrophages was relatively reduced, and the Mu vector restored the above inhibition. These results imply that TUSC7 is a specific target gene for miR-23b (Figure 3).

**Figure 4:**
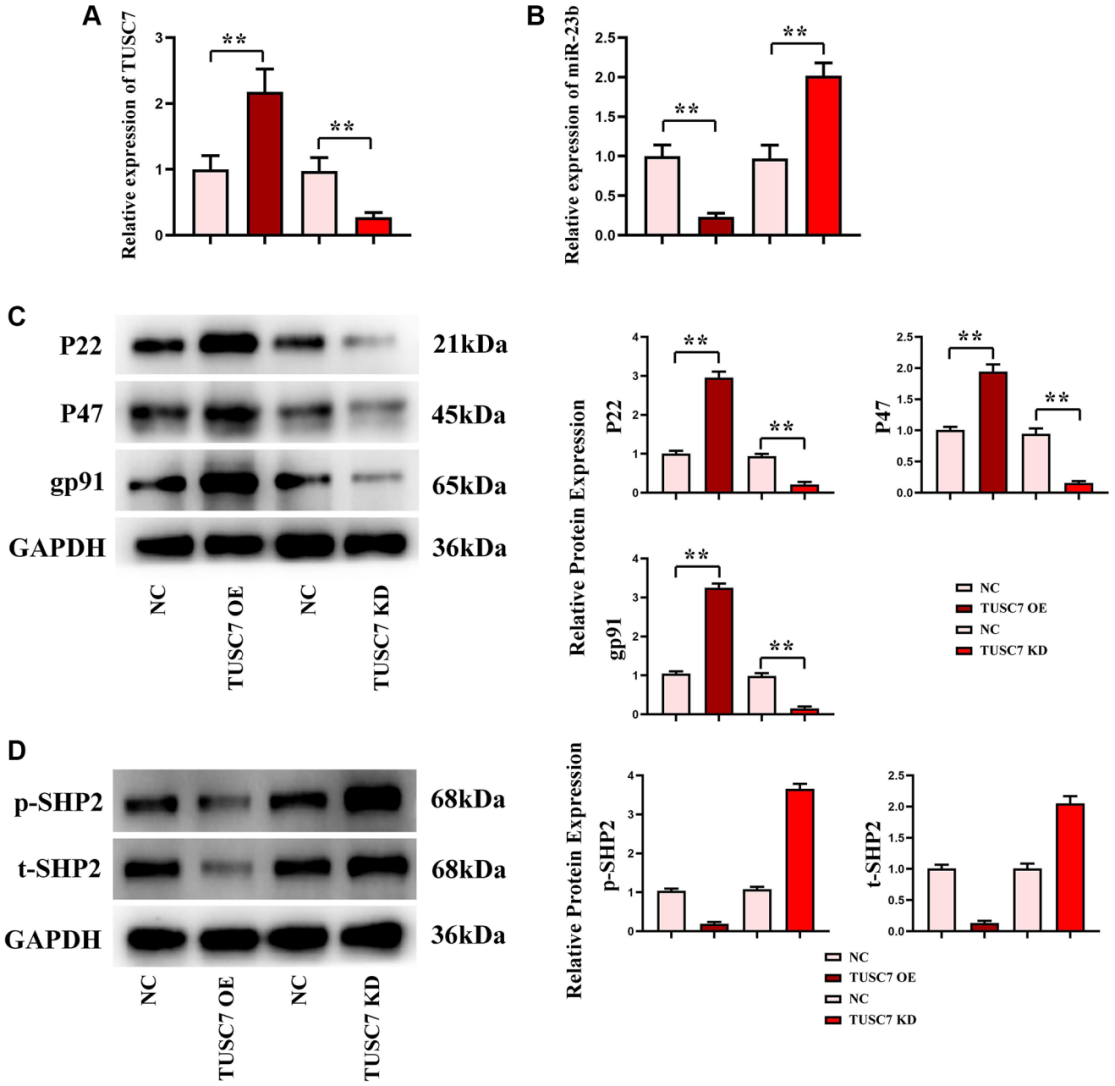
**Transfection efficiency detected by real-time quantitative PCR, oxidative stress and SHP2 phosphorylation levels in peritoneal macrophages.** (**A**) Data statistics of TUSC7 expression in peritoneal macrophages in each group. (**B**) Data statistics of miR-23b expression in peritoneal macrophages in each group. (**C**) Protein bands of expressions of oxidative stress-related proteins P22, P47 and gp91 in peritoneal macrophages. (**D**) Data statistics of SHP2 phosphorylation level in peritoneal macrophages. ^**^p < 0.01.

The results of real-time quantitative PCR showed that compared with the NC group, the expression level of TUSC7 in the TUSC7-OE group was significantly increased, the expression level of miR-23b was significantly reduced (*p* < 0.01), however, the expression level of TUSC7 in the TUSC7-KD group was significantly reduced, and the expression level of miR-23b was significantly increased, indicating that TUSC7 could inhibit the expression of miR-23b (Figure 4A, 4B).

### Transfection efficiency and effects of TUSC7 on oxidative stress and SHP2 phosphorylation in peritoneal macrophages

The results of real-time quantitative PCR revealed that in TUSC7 OE group, TUSC7 had a significantly higher expression and miR-23b had a markedly lower expression than NC group (*p* < 0.01), while in TUSC7 KD group, TUSC7 had a significantly lower expression and miR-23b had a significantly higher expression than NC group (*p* < 0.01), indicating a successful transfection. Furthermore, the results of Western blotting showed that compared with NC group, the expression of P22, P47 and gp91 in TUSC7 OE group was significantly increased, and the expression of p-SHP2 was significantly reduced. Meanwhile, the expression of P22, P47 and gp91 in the TUSC7 KD group decreased significantly, and the expression of p-SHP2 was significantly increased (*p* < 0.01). It can be inferred that TUSC7 can enhance oxidative stress in CRC cells and reduce phosphorylation of SHP2 (Figure 4C, 4D).

### TUSC7 could promote M2 polarization of macrophages

**Figure 5:**
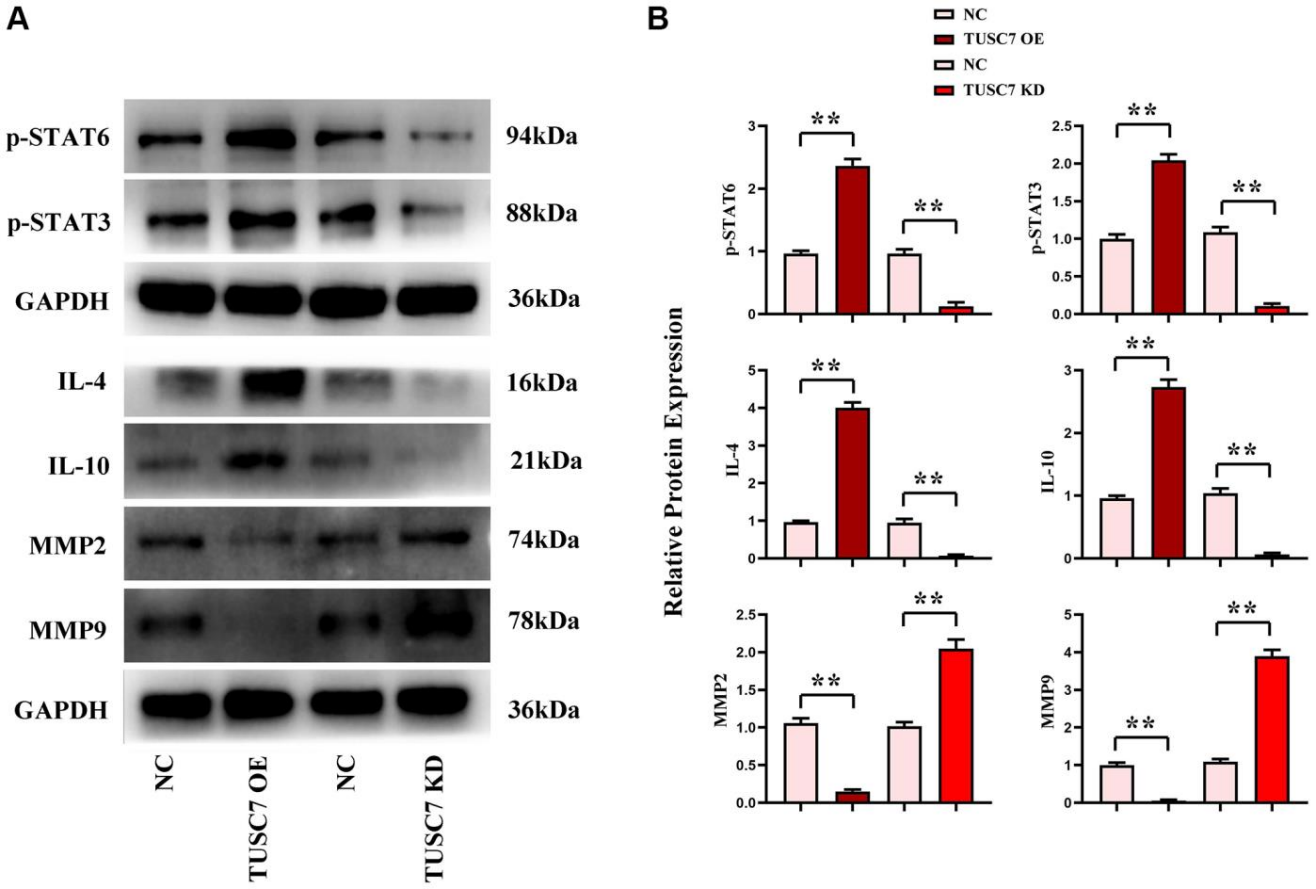
**Expressions of STAT3/STAT6 signaling pathway-related proteins in peritoneal macrophages.** (**A**) Protein bands of STAT3, STAT6, IL-4, IL-10, MMP2, and MMP9. (**B**) Data statistics of protein expressions of STAT3, STAT6, IL-4, IL-10, MMP2, and MMP9. ^**^p < 0.01.

The results of Western blotting showed that TUSC7 OE group exhibited significantly higher expressions of STAT6, STAT3, IL-4 and IL-10 and significantly lower expressions of MMP2 and MMP9 in comparison to NC group (*p* < 0.01), whereas TUSC7 KD group had the opposite results (*p* < 0.01). These results manifested that TUSC7 could promote the M2 polarization of macrophages and suppress the MMP expressions through the STAT3/STAT6 signaling pathway (Figure 5).

### TUSC7 could suppress the migration and invasion of CRC cells

**Figure 6:**
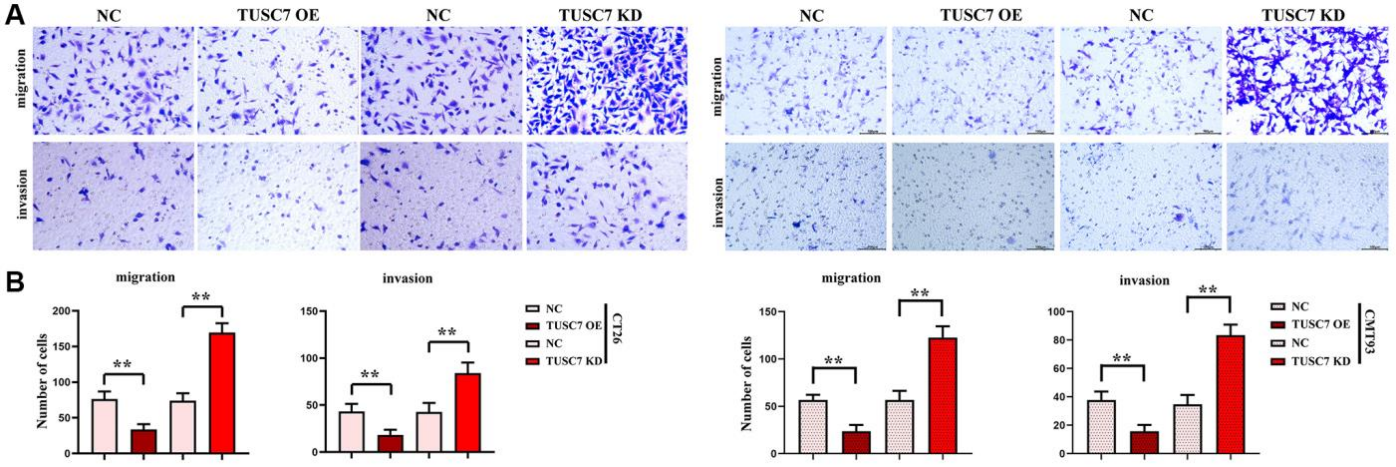
**Migration and invasion of CT26 cells detected by Transwell assay.** (**A**) Results of migration and invasion assays. (**B**) Data statistics of migration and invasion assays. ^**^p < 0.01.

In the Transwell assay, there were significantly more migrating and invading cells in TUSC7 OE group and obviously fewer migrating and invading cells in TUSC7 KD group than NC group (*p* < 0.01), indicating that TUSC7 can inhibit both migration and invasion of CRC cells (Figure 6).

### TUSC7 could suppresses the proliferation of CRC cells

**Figure 7:**
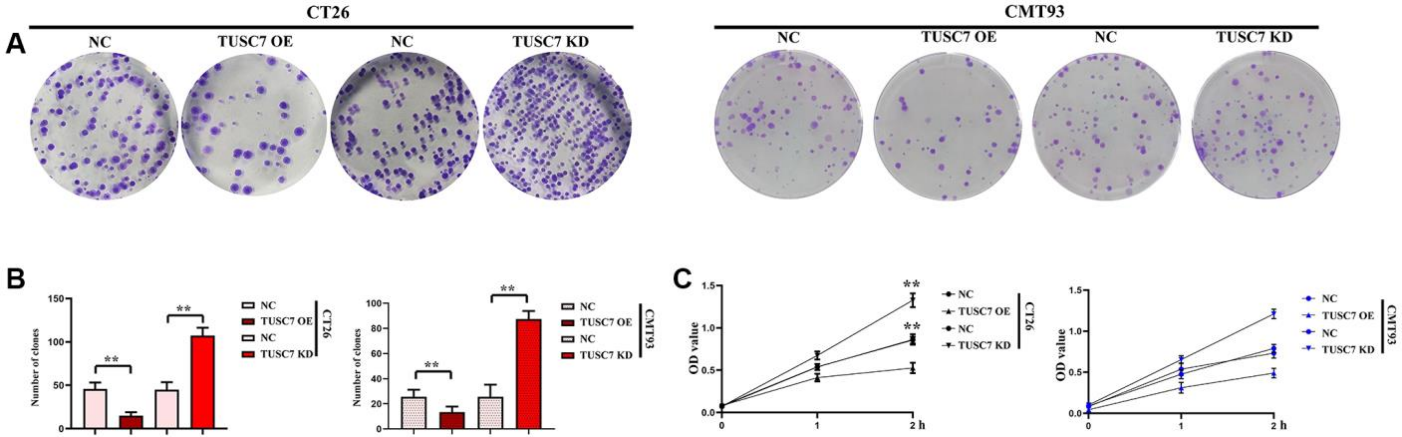
**Cell proliferation was detected by monoclonal formation assay and CCK8 assay.** (**A**) Results of monoclonal formation assay. (**B**) Data statistics of monoclonal formation assay. (**C**) Data statistics of CCK8 assay. ^**^p < 0.01.

The results of monoclonal formation assay showed that the number of colonies was significantly raised in TUSC7 OE group but evidently reduced in TUSC7 KD group compared with that in NC group (*p* < 0.01). In CCK8 assay, the OD values at 24 h and 48 h were significantly higher in TUSC7 OE group than those in NC group, but they were significantly lower in TUSC7 KD group than those in NC group (*p* < 0.01) (Figure 7).

## DISCUSSION

Despite recent advances in treatment, CRC remains one of the most aggressive malignancies, characterized by rapid recurrence and early metastasis, and it still relapses in many patients nowadays. Recent studies have shown that more than 75% of human genomes can be transcribed into RNAs, but only about 1.2% of these RNAs are translated into proteins. A large proportion of non-coding RNAs may have the potential to play important and intricate physiological and pathological roles [20–23]. Moreover, lncRNAs are implicated in cell proliferation, apoptosis, autophagy, invasion and migration. After being studied in recent years, the association between the function of lncRNAs and disease has been determined. LncRNA TUSC7 was first identified in osteosarcoma by Pasic et al. in 2010 [24], and it has been reported in some literature since then. TUSC7 serves as a potential tumor suppressor in some malignancies, including CRC, non-small cell lung cancer, hepatocellular carcinoma and gastric cancer [11, 12].

Increasing numbers of studies have shown that lncRNAs function as ceRNAs to regulate miRNAs in tumor progression. According to earlier studies, TUSC7 can serve as a tumor suppressor gene in several tumors via inhibiting the expression of such miRNAs as miR-10a, miR-211 and miR-23b. While this study has focused on the inhibitory effect of TUSC7 on miR-23b. It has been shown that TUSC7 was downregulated in gastric cancer tissues, and overexpressed TUSC7 reduced the growth of gastric cancer cells *in vivo* and *in vitro* by inhibiting the expression of miR-23b. Similarly, the expression of TUSC1 was lower in CRC cell lines (HT7, SW29, SW480 and DLD-620) than that in normal colonic epithelial cell lines (FHC) [25–27]. Among 40 cases of CRC samples, 7 cases had a decreased expression of TUSC5 compared with that in para-carcinoma tissues. The expression of TUSC7 in CRC samples was also lower than that in paired non-cancerous tissues. Additionally, TUSC7 was expressed less in high-grade (Dukes C and D) CRC samples than it in low-grade (Dukes A and B) CRC samples. The overall survival rate was lower in patients with a lower TUSC7 expression than that in patients with a higher TUSC7 expression. Both proliferation and invasion of CRC cells were restrained by increased expression of TUSC7 [28–30]. TUSC7 can be found to inhibit the metastasis and progression of CRC cells by observing the abdominal circumference of mice. All these results suggested that TUSC7 acts as a tumor suppressor gene in CRC.

More importantly, in this study, dual luciferase assay showed that TUSC7 could specifically inhibit miR-23b. The results of real-time quantitative PCR showed that CRC cells had a lower expression level of TUSC7 and a significantly higher expression of miR-23b than peritoneal macrophages. It was found by *in vitro* cell assays that TUSC7 could increase the expressions of P22, P47 and gp91 in CRC cells. It can be seen that TUSC7 can not only enhance the oxidative stress and SHP2 phosphorylation in CRC but also increase the expressions of IL-4 and IL-10, promote the M2 polarization of macrophages and suppress the expressions of MMP2 and MMP9 through mediating the STAT3/STAT6 signaling pathway. In addition, the results of Transwell assay revealed that TUSC7 could inhibit the migration and invasion of CRC cells, thereby suppressing the metastasis of CRC. The ability of TUSC7 to inhibit the proliferation of CRC cells was confirmed in monoclonal formation assay and CCK8 assay.

The above results have confirmed that TUSC7 is down-regulated in CRC cell lines, and overexpressed TUSC7 can suppress the proliferation, migration and invasion of CRC cells by inhibiting the expression of miR-23b. Therefore, TUSC7 may become a new therapeutic target for CRC.
